# Book Reviews

**Published:** 1806-02

**Authors:** 


### CRITICAL ANALYSIS
### OF THE
### RECENT PUBLICATIONS
### ON THE
### DIFFERENT BRANCHES OF PHYSIC, SURGERY, AND MEDICAL PHILOSOPHY.

---

*Observations on the Nature and Cure of Gout, and Nodes of the Joints; and of the Influence of certain Articles of Diet, in Gout, Rheumatism, and Gravel; by* James Parkinson, *Hoxton,* 8vo. pp. 174. 1805.

Whatever comes from the respectable pen of Mr. Parkinson, must be entitled to notice: and when we are informed in the preface, that he has himself been an arthritic, and found relief from the plan he proposes, we cannot but peruse his labours with peculiar interest. In doing this, however, we shall not be backward in giving our opinions with a freedom which cannot offend the author; and, we trust, may serve the interest of science.

In the first Chapter, Mr. P. describes the character of gout; enquires into the proximate causes; adverts to the discoveries from Dr. Woollaston's experiments, and to the dissection described by Mr. Watson in Medical Communications; concluding with an enquiry respecting the existence of a peculiar acrimony in this disease.

"Gout," says our author, "is an hereditary disease, chiefly affecting, with pain and inflammation, parts possessing a ligamentous or tendinous structure. It most frequently attacks the joints, and particularly those of the hands and feet. It sometimes also manifests itself, by painful affections of internal parts, which often alternate with the affections of the limbs. It deposits, on the parts parts which it affects, a concrete saline substance, which is sometimes accumulated in considerable quantities, particularly on the joints of the fingers and hands.

"The different forms in which this disease appears, have rendered it necessary to divide it into regular and irregular gout. In the former, the attacks of which are known by the denomination of regular fits of the gout, one or more joints of the extremities become inflamed, painful, and tender, and frequently in an exquisite degree. A symptomatic fever, proportioned to the degree of pain and inflammation, with evening exacerbations, accompany the other complaints, which distress the patient for uncertain periods, sometimes for several weeks. When the fit goes off, the joints, which have been the seat of the disease, are always found to have become rigid and inflexible, in proportion to the degree in which the disease has existed in them; frequently remaining enlarged, and incapable of free motion, for a considerable time. On the other hand, the patient, at the same time, experiences so perfect an exemption from disease, as generally to lead to the opinion, that the fit has occassioned the most salutary changes in the system.

"In the irregular gout, the affection of the joints is much less confined than in the former. Sometimes it leaves the joints at first attacked, and fixes on some distant part; and sometimes, after harrassing the patient, by making a circuit including almost every joint of the extremities, the fit is terminated by a renewed attack on the part first affected. In some cases, the disease quits its situation in the extremities for a time, and occasions symptoms of a very alarming nature, by its attack on some internal part; this also abating on the return of the disease to the part which had been first attacked: this is termed, retrocedent gout. In other cases, in which there exist the most evident marks of a gouty diathesis, no affection of the extremities takes places, but complaints of a very anomalous kind shew that some internal part is under the influence of this disease: these may be regarded as cases of misplaced gout. A want of power and tone in the system appears to accompany both these states of gout.

"The proximate cause of gout appears to be—a peculiar saline acrimony existing in the blood, in such a proportion, as to irritate and excite to morbid action, the minute terminations of the arteries, in certain parts of the body."

In pursuing this inquiry, inductions are formed from the acidity evolved from the stomach from the acescent drinks taken by the wealthy, who are the principal subjects of gout, from the superabundance of phosphate of lime, and, probably, other extraneneous matters in the urine of older people after the process of ossification has been completed; and from the great quantity of fluid, which Dr. Woollaston found necessary for the solution of gouty calculi, which proves its constant disposition to precipitate.

In

In the next Chapter, the author enters on the remote causes. The principal of these are, hereditary disposition; indigestion; errors in non-naturals; intemperance; different effect from different fermented liquors; circumstances preventing the escape of morbid acid. On this we would wish to remark, that when hereditary disposition is considered as a remote cause, it ought not to be confounded with those afterward enumerated, which are altogether accidental, or such as may be prevented. We should rather consider hereditary predisposition (a term we think preferable to a word often applied to other conditions of the body) as an original organization, rendering the constitution, or certain parts, susceptible of the impression from the remote causes. It is well known, that these same causes produce different effects, according to the original organization, and that such organization depends on causes, we can neither trace nor controul, nor even know, but by the effects of the remote causes.

Among these causes, Mr. P. considers wine, and still more, cyder, as the most considerable. Beer, he conceives innocent, till it has been kept long enough to acquire a degree of acidity. A long quotation follows from Dr. Cadogan, which, we suspect, our author would not be ready to adopt as his own; for, if fermenting liquors of any kind, and bread, are so dangerously acescent, it is difficult to conceive, why women, and such of the labouring class whose occupations are sedentary, are not universally gouty, as they are not less subject to all the circumstances which prevent the escape of the morbid acid than the wealthiest arthritic.

The third Chapter contains an examination of the symptoms and their agreement with the supposed proximate cause. In this the author enumerates most of the symptoms, and shows, that they may for the most part, be attributed to the evolution of acidity, and of its retention from the cessation or diminution of any customary discharge. In this chapter, much is insisted upon from the frequent occurrence of gout and urinary calculi in the same constitutions. This appears to us an error originating with Sydenham, and adopted from him by every succeeding writer. If it is not an error, it is, at least, not supported by a sufficient number of facts to be considered, as it usually is, among those data on which presumptive reasoning may be supported.

In the fourth Chapter are contained: the diagnosis; difference between gout and rheumatism; anomalous complaints apparently dependant on gout. We are not satisfied with all the inferences our author would draw from the facts he produces in this part of his reasoning. It is well known, that inflammation of any kind will shift from one part to another, and also that diseases will succeed each other in the same constitution: that these inflammations will produce effects according to the parts affected; and the diseases, whilst one supercedes the other, seem totally unconnected with each other, not only in their symptoms, but in their supposed remote causes.

The fifth Chapter contains some very useful practical remarks on particular affections of the joints, apparently dependant on the same state of the system as that which produces gout. Dr. Haygarth has given a very accurate description of this disease, nor has it been overlooked by the accurate Heberden.

As there are some differences in the descriptions of these Gentlemen, and also in their opinions, as to the gouty origin of the complaint, it is much to be regretted, that Mr. Parkinson did not make use of a chemical test to ascertain the component principles of those enlargements which form the character of the disease in question. As the description here offered is very minute, we shall transcribe it, that our readers may compare it with those of Drs. Haygarth and Heberden.

"It generally first manifests itself in one of the last joints of the fingers; the ends of the bones forming the joint become slightly painful and tender, and a small degree of enlargement is at first perceived. The swelling, with the tenderness and pain, gradually increase; so that, at different periods, in different persons, but generally in about three months, the pain and enlargement occasion considerable inconvenience. Myriads of minute hot points sometimes seem to be piercing the bone, whilst at other times a stinging sensation pervades the tumefied part; the pain being considerably increased by the least pressure. The motion of the joint becomes so impeded by the enlargement, that the merely closing of the hand, in its ordinary employments, produces a very considerable degree of pain.

"As the swelling continues to enlarge, a very slight degree of redness comes on, and sometimes threatens suppuration, which, however, very seldom ensues. Sometimes this inflammatory state, after continuing a certain time, varying much in this respect in different persons, at length subsides, when the mobility of the joint is found to be much diminished, and the swelling increased in size and hardness, but much less painful and tender. In this state it sometimes continues, with the exception of a very gradual increase of the size and hardness of the tumour, and consequent injury to the motion of the joint, during the remainder of life.

"Within a little time, perhaps two or three months, of the appearance of this first swelling, some of the first joints of the other fingers become affected in a similar manner, and pass through a similar course: and thus most of the other finger joints become the seat of this malady, and undergo the morbid changes just described.

"As the mischief advances in the hand, proofs of the general influence of a peculiar morbid state are evinced, in some of the larger joints, particularly in the wrists, the elbow, the ankle, and knee joints. But even when the larger joints are attacked, it is not to be ascertained, in the living subject, whether the seat of this malady is in the more prominent parts of the ends of the bones, which form the joint, or of the periosteum, just before it separates to give a covering to the capsular ligament, or in the ligamentous parts alone. Sometimes extreme tenderness, on pressure being applied, shews that the os calcis, or its immediate investiture, partakes of the mischief. This tenderness, which, in the first of the morning, will hardly allow the foot to rest on the ground, diminishes after the pressure has been repeated, during walking, for about half an hour. Sometimes the tenderness, accompanied by a slight degree of enlargement, exists at the back part of the os calcis; and in one case a knotty seam-like hardness was discoverable in the *tendo achillis*, which very much impeded the walking. A hardness somewhat similar, with an indentation and contraction affecting even the integuments, is also sometimes observed in some of the flexor tendons of the fingers, in those who possess the diathesis here described. When this is the case, the corresponding finger will always be found firmly contracted, in proportion to the injury which the flexor tendon has experienced.

"Frequently the bones of the feet become so affected, as to occasion great difficulty and pain in walking, which is only performed, in a manner, which renders the crippled state of the parts very evident; giving the idea to the patient of the metatarsal bones, which form the arch of the foot, being crushed together by the pressure of the body.

"After some time, most of the joints, and, with the rest, those of the spine, partake of the prevailing disposition to rigidity; so that at last the flexibility necessary for performing the most simple offices in life is lost. Thus crippled, the unhappy sufferer sinks under his calamity; his various incapacities, proceeding from his inability for motion, giving the idea of his being prematurely afflicted with the decrepitude of old age.

"The persons who appear to be most liable to this complaint, are those to whom its injurious effects must prove most particularly afflictive. The labouring poor, whose hands are their only means of support, appear to be the most frequent sufferers by this malady. A slight and transient injury to the hand is, indeed, a serious injury to the poor: but a disease which thus entirely destroys its powers, renders the situation of its victims truly deplorable. They toil on, depressed by observing the daily diminution of their ability for laborious exertion, and are at last mournfully obliged to submit to receive from charity, that support, which their hands can no longer procure them. The examination of the inmates of those houses which receive the parochial poor, will generally shew sufficient proofs of the prevalence of this malady. Many will be found driven thither who still possess a considerable portion of constitutional strength, but who, thus maimed, are entirely deprived of that blessing to an independent spirit, the power of supporting themselves by their own exertions.

"The misery which this complaint sometimes inflicts, is thus enlarged enlarged upon with the hope of exciting the attention of medical men towards it and of inducing them to make known, in their respective circles, those observations which may be likely to prove beneficial in mitigating evils, which must be so severely afflictive to the poor.

"The particular enlargements of the ends of the bones here described, differ sufficiently from those which take place in scrophula, to allow the distinction to be very easily made: they occur much later in life than the latter; the tumours never acquire that magnitude, nor that soft and pulpy feel; nor does the skin possess that glossiness and redness which are observable in scrophulous tumours; neither do they, except very rarely, terminate in suppuration. They differ from those tumours of the joints which proceed from external injury, and which generally accompany a carious state of the bones, in the pain and tenderness, as well as the inflammation and tumefaction, existing in a much less degree than in those. Whether they differ essentially, or only in degree, from those tumours which are formed by the gouty concretions, does not appear to be certain. The first appearance of the chalk-stone, as it is termed, is not unlike that of these tumours; but in general the gouty concretion becomes much sooner pointedly prominent, the morbid matter is more rapidly deposited; the integuments also become pointed and shining, and soon become extremely thin and then ulcerated, allowing the gradual escape of the deposited matter. But in the tumours, to which our attention is here particularly directed, the tumefaction, instead of soon becoming prominent, preserves the general form of the end of the bone, thus enlarged, the integuments undergo but very little change, and, as has been already observed, suppuration seems rarely to take place.

"The only instance, at present known to the author, in which this species of tumour had proceeded to suppuration, was in a female about fifty years of age, a maniac, and in a very infirm state. Suppuration had taken place on five of the finger joints, and in one knee, in every one of which the ends of the bones were so carious, as would have rendered amputation necessary, had she not been in that reduced state which forbad it. The derangement of her mind was such as prevented her furnishing any account of the origin of these tumours, the inflammation and suppuration of which was attributed to her having been constantly exposed to considerable cold during the preceding winter."

The mode of cure which the author found effectual in himself and some others, was by the application of leeches; by producing a constant moisture on the part; by surrounding it with some moderately adhesive plaster; by a proper attention in avoiding as much as possible ascescent foods, to lessen the quantity of acidity in the system; and, lastly, by the exhibition of soda to neutralize what acidity may be formed.

In the sixth Chapter, the author enters into an enquiry concerning concerning the indications of cure in gout. These he states—to prevent the formation of the morbid acidity; to remove and correct that which already exists; and to repair the diminished strength of the system. A recollection of the proximate cause will at once suggest the proper remedies. The author illustrates his mode of cure by some very pointed cases, and the practice is supposed to be further indicated by the effect of alkalis in nephritic diseases, and even in chronic rheumatism. Mr. P. seems to think, that the advantages Dr. Dawson found from the use of volatile tincture of guaiacum in gouty cases, were very much to be ascribed to the alkaline, as well as the stimulating property of the remedy.

The next Chapter, on the mode of treatment during the paroxysm, contains but little novelty. The author has in this place introduced Major Rook's well known, but we ought to add, solitary case, in which gout instantly gave way to a spontaneous vomiting of acid matter. From hence, and other well-known facts, he insists much on the necessity of attending to the stomach during the whole paroxysm. In this, and the other remedies proposed, nothing very new is suggested.

The last Chapter contains Remarks on Dr. Kinglake's Theory; on Retrocedent Gout; and on the danger of applying cold water. This is illustrated with some very interesting cases; but though we have never given a decided preference to Dr. Kinglake's practice during the whole controversy, which, we trust, we have conducted with impartiality; yet, that impartiality obliges us to allow, that we do not think Mr. Parkinson's cases will admit all the inferences he would draw from them. That active inflammation suddenly suppressed in one part may show itself in another, seems necessarily to follow that plethoric state of the constitution from which spontaneous inflammation may arise. We have therefore always given it as our opinion, that these immersions in cold water should be accompanied with general evacuations. But those chronic diseases, which Mr. P. ascribes to the retrocession of gout, might, we conceive, happen from any occasional debility in a gouty constitution which should prevent that active inflammation necessary to form acute gout. However, by his own case and manner of treating it, we may infer, that chronic gout, or symptoms which cannot easily be distinguished from it, may be relieved, and perhaps cured, by a well regulated diet, and the occasional exhibition of alkalis.

Before we take our leave of this useful little performance, we would suggest a doubt, whether inflammation in those parts, which are the seat of gout, is not necessarily attended with such a secretion as is considered as characteristic of the gouty action; and whether the abstainence from fermented liquors, and even the exhibition of alkalis, may be of any other use than that of preventing inflammation. The consideration is highly interesting, and we think cannot be in better hands than Mr. Parkin-

 son son's, who has leisure and genius to pursue it, and whose reports will be received with such confidence, as should encourage his perseverance and frequent communication.

---

*Observations on the Utility and Administration of Purgative Medicines in several Diseases.* By JAMES HAMILTON, M. D. Fellow of the Royal College of Physicians, and of the Royal Philosophical Society; and Senior Physician to the Royal Infirmary. 8vo. pp. 320. Edinburgh, 1805.

THE reputation of Dr. Hamilton, as a practical physician, is too well known to require any comment here; and it always gives us much satisfaction, when we find such men as these, offering the result of their observations to the public. From them we expect certain aphorisms, which may be referred to by younger practitioners; instead of fine spun theories, many of which vanish as soon as they appear.

Though the present performance does not claim any great pretensions to novelty, yet it abounds with valuable remarks; and, we must conclude, it is not without some necessity, that it issues from the source to which we owe it. Probably in the North, greater prejudices against purgative medicines may prevail than in the South. But let us first take a view of the work, that our readers may be enabled to follow us in our remarks.

The first Chapter contains general observations on purgative medicines. After some remarks on the changes to which the practice of physic must always be liable, the author continues:

"I make these observations as an apology, if one is necessary, for my having occasionally deserted the usual track, which has been pointed out by men of consideration in practical medicine. I have deserted this track, more particularly in what relates to the administration of purgative medicines. I have been led to consider this subject minutely, from a habit of attending to the means of snpporting, and, when necessary, of restoring the healthy action of the stomach, and intestinal canal; which action is of great importance, and which is liable to be disordered, and in this state, to produce much distress in various diseases.

"In the course of the following observations, when I call in question the opinions of respectable authors, I trust I shall speak with that deference and caution, which I feel to be due to them; being well aware how apt we are to take erroneous views in discussions that are purely theoretical.—And when I propose those changes in practice, which experience has taught me to be useful, I will do so with a confidence proportionate to that experience upon which my opinions are founded. Nevertheless, I beg it to be understood, that I respectfully submit the changes which I suggest, to the consideration of my professional Brethren; and request, that, after a full trial, they will give a candid decision on their merits; for the judgement of the public is the test, by which, which, I am sensible, they must ultimately be confirmed or rejected.

"The importance of the functions of the stomach and intestines is well known, and universally admitted. By means of these functions our food is digested and assimulated, and is carried, under the form of a nutritious fluid, into the system. Besides, the power of sympathy connects the stomach and bowels with many other parts of the complicated animal machine; and strengthens the influence which these organs maintain over the comfort, the health, and the life of every individual. Hence it is obvious, that disorders of the stomach and bowels must greatly affect the system at large; and that, in proportion to the degree and duration of these disorders, the affection of the general habit will be more or less serious and afflicting.

"There is certainly nothing new in the observation, that the constipated and loaded state of the intestinal canal, is a common cause of general bad health. But when I go the length of saying, that this state generally accompanies and aggravates the other symptoms of fever; that it is also the immediate cause of certain disorders peculiar to children and young people; I am aware that I advance opinions, in which there is a considerable degree of novelty; but in which, I trust, the following sheets will satisfy the medical reader, that there is also, at least, an equal degree of soundness."

The subject is continued a few pages further, bnt nothing occurs of sufficient importance particularly to arrest our attention.

The next Chapter, is on the utility of purgative medicines in typhus fever. The most useful observation in this, is, that the indiscriminate use of emetics in the beginning of these fevers, so generally adopted by the disciples of a once celebrated professor, is discouraged or shewn to be often unnecessary. The author found great advantages from brisk purgative medicines in a fever which spread from the French prisoners, and also from the crew of a merchantman at Leith. In these, he discovered, that his antimonial medicine was only efficacious when it proved purgative. This induced him to ensure that effect, with more certainty, by a proper combination with other ingredients; and the event answered his expectation. Since that time, he has seen no reason to alter, but every thing to confirm his practice. These remarks conclude in the following words:

"Accordingly, it is now some years since I have left off, almost entirely, the practice of ordering emetics and glysters in fever. I trust to a purgative, to ensure a regular alvine evacuation. For this purpose, however, a daily purgative is not always required. Thus, avoiding the harrassing distress, which generally accompanies the operation of an emetic given to patients in a state of fever; as well as the trouble and fatigue which the exhibition of glysters occasions; I think I conduct the treatment

 of of typhus fever to a favourable issue, with more certainty, and with the greater ease and comfort of the patient.

"This practice, which I have found useful, and which respects only the state of the intestinal canal, supersedes by no means, usual attention to the various other means of cure, employed in fever. I am even ready to allow, although I exclude emetics and glysters from my general practice in typhus, that peculiar circumstances may, occasionally, make both the one and other necessary.

"I cannot, however, omit remarking, that for some years past, I find wine less necessary in fever, than I formerly thought it was. This may be owing to the fever which has prevailed of late being less malignant than it was some years ago; or to the effect of the purgative medicines which I have employed, and which may obviate symptoms of debility, as well as remove them.

"If this is a just view of the case, the plain inference is, that the employment of purgative medicines, to preserve a regular state of the belly, does not increase the debilitating effects of fever. This doctrine, I know, is contrary to the opinion generally received; but I am confident, that it is consonant to the fact.

"The object to be attained, is the complete and regular evacuation of the offensive feculent matter collected in the bowels, in the course of fever. Within this limit, the practice is safe and salutary. Of this I am assured, that I have had much satisfaction in the prosecution of it; and have not in a single instance, had occasion to regret any injury or bad consequence proceeding from it. For I am not an advocate for its being carried to the length of exciting unusual secretion in the cavity of the intestines, or of procuring copious watery stools. Such indeed, while they are not requisite, might increase the debility so much and so justly dreaded.

"In further recommendation of the practice, I observe that it is conducted with ease, and a tolerable degree of certainty. The precise effect of purgative medicines, may not, in every instance, be altogether under command; but in general it is so, if, to a little experience, we join a previous knowledge of the peculiarities in particular constitutions. At any rate, the subsequent doses of purgative medicines, and the repetition of them, will be regulated by the effect of preceding ones.

"It is of importance, to consult in all respects the quiet and comfort of patients, in fever. On this account, the exhibition of purgative medicines should be so timed, that their effects may be expected during the day, when proper assistance can be best procured to the patient.

"The purgative medicines which I have chiefly employed in fever, are calomel, calomel and jalap, compound powder of jalap, aloes, solutions of any mild neutral salt, infusion of senna, and sometimes the two last mentioned medicines conjoined."

The

The second Chapter is on the utility of purgatives in scarlatina. The author is disposed to consider cynanche maligna as the same disease, and advises a similar exhibition of purgative medicines in each. As, however, his principal design is rather to relieve the intestines from any injurious accumulation, than to lessen the strength of the patient, we apprehend, few of our readers will be disposed to differ with him.

Nor is it likely many objections should be made against the exhibition of purgative medicines in that species of " marasmus, which appears in childhood and early youth." The advantage of full doses of calomel are now generally admitted. We shall therefore content ourselves with transcribing a single passage, which, though not altogether new, contains a caution, that cannot be too often impressed on practitioners of every age and rank in the profession.

" While," says the author, " I thus give appropriate purgative medicines, I find it necessary, in order to have full information of their effects, to inspect daily what is passed at stool. The smell and appearance of the fæces are a criterion of the progress we make in the cure, and direct the farther administration of the purgatives. This inspection is the more necessary, as we cannot expect the information we want from our little patients; and we will often look for it in vain from the attendants, whose prejudices, and whose ignorance of our views, prevent their seeing the propriety of the enquiry.

" During the prevalence of the disease, the fæces are dark, fetid, and varying from a costive consistence to that of clay, and are often fluid; and such they appear upon the first exhibition of the purgative medicines. I observe that the recovery of the sick keeps pace with the return of fæces of natural colour, form, and smell; a change which the repetition of purgatives does not fail to produce."

Some useful remarks are added on the indolence of many practitioners in imputing so many of the complaints in children to worms. Though this error is gradually losing ground, we trust, the remarks made by this judicious writer will not be without their use.

Dr. Hamilton is of opinion, that marasmus either precedes or accompanies hydrocephalus, epilepsy, and other formidable diseases. This is certainly true, and we doubt not, that he has often been successful in relieving them by strong purges. We can say the same of our own practice; but in these cases, we have directed our views to relieve a kind of chronic or habitual plethora, rather than the removal of accumulated fæces. This suggestion is not intended to lessen the value of our author's remark, which, we doubt not, is founded on close observation.

The next Chapter is on the utility of purgatives in chorea sancti viti. In this, it is shown, that however mistaken the illustrious Sydenham might be in his theoretic notions of this disease, ease, yet his practice was perfectly conformable to what is here proposed. It is much to be regretted, that the erroneous theories of that invaluable author, have in so many instances entailed upon us a practice contrary to his own, and for which we have, in many instances, invented theories little better than his. The cases given by our author, prove the justice of his opinions beyond any question, but we cannot admit it as a necessary inference, nor do we conceive it will be expected, that chorea never arises from any other causes than those here assigned.

The following Chapter on chlorosis is particularly deserving notice. This disease, though more common in the North, and among young females who are ill fed and too sedentary, is, however, known in all climates, and in every rank of life. It has been too common to impute it, altogether, to amenorrhœa, and consider chalybeates as the only remedy. It must be confessed, that however general the success of these remedies may have proved, they have often failed; and we are under many obligations to Dr. Hamilton for pointing out other causes.

The sixth Chapter is on the utility of purgative medicines in one species of hæmatemesis or vomitting of blood, which the author describes in the following words.

"There is, says he, one variety of hæmatemesis which attacks females who are from eighteen to thirty years of age; and it rarely appears sooner or later than these periods, which I shall endeavour to illustrate.

"As I confine my attention to this variety, the observations which I am about to make, will not apply to hæmatemesis, which originates in organic affection of the stomach, and viscera connected with it, either as a constitutional disease, or the consequence of previous irregularities and intemperance. I have seen several instances of this vomiting of blood, the cure of which is doubtful in the extreme and difficult.

"The attack of the hæmorrhagy, of which I am to speak, is preceded by great languor and oppression, both about the chest, and the præcordia; and by a sense of fulness of the præcordia, by cough, dyspnœa, and sometimes by pain of breast; by loss of appetite, head-ach, vertigo, and disturbed sleep. The eye is dull, the countenance is expressive of much distress, the pulse is feeble, and the bowels are constipated.

"In this state of impaired health, a particular fit of sickness and nausea is the immediate fore-runner of the attack of the vomiting of blood. The blood vomited is sometimes florid, and, at other times, black and grumous. The quantity of blood brought up at one time, varies from a few ounces, to the quantity of a pound or more. The distressing symptoms are relieved by this discharge of blood; but are again aggravated, previously to the return of a similar attack.

"This disease, under the usual management, is of uncertain duration, and of unequal severity.

"The

"The time of life, at which this hæmatemesis takes place, and the circumstance of being peculiar to the female sex, have induced practitioners to imagine, that it is intimately connected with the menstrual flux; the suppression of which has been generally considered as the sole cause of the disease. It has been said to be a hæmorrhagy, vicarious of the menses.

"The high importance of the uterine system in the animal œconomy cannot be doubted; but the functions of this system are veiled in deep obscurity, and will not, perhaps, be at any time clearly understood. They have occupied much of the attention of the speculative enquirer; and ingenuity has been taxed, to invent theories in explanation of them, and of their influence, in health and in disease.

"The menstrual flux, the most obvious of the uterine phenomena, has afforded a wide field for discussion. It is interwoven with the opinions we entertain of almost every disease, to which the female sex is exposed. Its overflow, or its suppression, are the ready expounders of many symptoms; and the fruitful, though perhaps imaginary source of many diseases. This flux is a constant object of attention to females, who are, in general, well schooled, as to the importance and necessity of it.

"These theories of the schools, and these early impressions on the female mind, give a consequence to this subject, and force it upon the notice of the medical practitioner, who must subscribe to the general opinions respecting the menses, and seem to adopt them, although he may question, in some respects, the foundation on which it rests, and the conclusions to which it leads."

This subject is continued much further by our author, but we do not think it necessary to transcribe more than sufficient to show his opinions. We are ready to allow all he urges relative to these mistaken notions, but cannot help thinking that there are practitioners who think too lightly of this secretion, and its attendant irregularities. Enquiries on this subject lead to a knowledge of our patient's constitution, and enable us often to form prognostics with much more accuracy than we could otherwise do. That however this hæmorrhage of which our author speaks is in its cause unconnected with the uterine discharge we are satisfied, not only because, like him, we have seen it in females who have menstruated regularly, but even in males. It seems oftentimes similar to, or to arise from similar causes with the discharge of blood per anum. The method of cure proposed by Dr. Hamilton, is in either case very rational, as nothing so much relieves a plethoric state, or a disposition to form blood, as frequent brisk cathartics, especially if administered antecedent to the periods when we might expect the return of such complaints. However, the cases produced by our author are strongly marked in favour of his hypothesis, and deserve to be accurately attended to.

The last disease for which this work proposes the exhibition of purgatives is hysteria. On this subject the remarks are general, but but such as we hope will attract the attention of his readers and the faculty in general. The following we think cannot be too often, or too strongly enforced:

"I have thus endeavoured to accomplish what I proposed, by showing that purgative medicines may be used more freely than has been commonly imagined; and used, not only with safety, but with evident, and decided advantage.

"Here I must again solicit the reader's attention to two circumstances of great importance, in the treatment of diseases, by the use of purgative medicines. The first is, the regular and accurate examination of every alvine evacuation. The second is, the steady exhibition of the purgative medicine, so as to procure daily its full effect, during the continuance of the disease for which it is given.

"By the inspection, we ascertain the nature of the alvine discharge; a knowledge of which, together with a few other circumstances, enables us to form a probable conjecture, with regard to the duration of the ailment, regulates the strength of each dose of the purgative, and determines the frequency of the repetition of it. Without this inspection, we will be constantly deceived, through the ignorance or inattention of our patients, or of their attendants.

"By the second circumstance, the steady exhibition of the purgative medicines, we ensure the success of the practice, in the diseases under consideration. The puny and debilitated state of the sufferer may, on some occasions, excite alarm even in the breast of the practitioner; and the caprice of his patient, and the whims of relatives, may throw obstacles in his way. But these he must disregard; for unless he can suppress his own improper feelings, and overcome the unreasonable objections of others, he had better not adopt measures, which, to prove successful, must be conducted with decision and firmness. A contrary conduct will not avail; but, on the other hand, it will assuredly terminate in the vexation of the practitioner, the disappointment of the patient and relatives, and the discredit of that practice, which, from a conviction of its utility, it has been my wish and study to recommend."

The Appendix contains a number of valuable cases of the above, and other chronic diseases, illustrative of the practice proposed, and highly valuable, when we consider the opportunities Dr. Hamilton has enjoyed for so many years, and the character he has uniformly maintained for accuracy and fidelity.

Having thus, we hope, done justice to this performance, we shall take notice of a few lines in the Preface. Here our author thinks it necessary to apologize for appearing before the public; and after giving an account of the clinical part of the Edinburgh education, adds:

"A number of well informed young gentlemen, who attend the hospital, have become converts to the free exhibition of purgative medicines which they see me employ with so much advantage. By this means the peculiarities of my practice here, passed silently into the world, unexplained and unsupported by proofs and illustrations tions which it was in my power to bring forward; they have been partially noticed in one periodical publication and made the subject of hasty and mistaken criticism in another."

What other publication may have noticed this practice, we know not; but for ourselves, we trust Dr. Hamilton will not accuse us of any partial notice of HIS "peculiarities of practice." The cases we met with were described by one of the young gentlemen he speaks of, and in a manner much too superficial to enable us to form those important decisions which the work before us induce. If, however, the partial notice in another publication, and the mistaken, we will not admit hasty, criticism in our own, have been the cause of the present production, we shall readily bear our share of the blame, and hope that the reception of his first labours will prove a sufficient encouragement for the author to enlarge them hereafter.

---

*A Practical Account of a Remittent Fever, frequently occurring among the Troops in this Climate.* By THOMAS SUTTON, M. D. of the Royal College of Physicians, London. Octavo, pp. 42, Canterbury, 1806.

"The object of this publication," we are informed, "is to give a compressed clinical account of a Remittent Fever, which the author has had repeated opportunities of investigating, while he was employed as Physician to the Forces; and he flatters himself, that he has been enabled to give some useful information on the nature of a disease, of frequent occurrence, among the military in this climate, during the cold months of the year."

Having premised thus much, Dr. S. next gives his reasons for suspecting the disease to be contagious:

"It has seldom happened, that officers belonging to regiments, in which this disease has occurred, have been infected; though the medical attendants of the sick, and servants attached to the regimental hospitals, have very rarely wholly escaped, and, in some instances, the whole of them have taken the disease.

"While patients, labouring under this fever, were in Deal General Military Hospital, (though not over crouded) where ventilation, fumigation, and cleanliness were much attended to, the medical mates and hospital servants, very rarely, remained long uninfected.

"The disease prevails to no considerable extent, except among men in barracks, and in confined and crouded situations.

"It has been observed to attack great numbers of one regiment, while another, under the same external circumstances, and within the same barrack wall, has remained, for some time, free from it.

"This fever has not been propagated, to any considerable extent, in the neighbourhood of those regiments, which have been attacked by it.

"These

"These circumstances prove, that the cause of the disease acts in a very confined sphere, and totally exclude the idea, that it is produced wholly by the qualities of the air, by the season, or any common surrounding source of unhealthiness; but the inductive proofs seem strongly to imply that its exciting cause is contagion."

We have copied thus much from the work, because we could not compress the author's meaning in fewer words. We might say the same of the "History of the Disease" which follows, and which we have perused with much pleasure several times over. Most of the symptoms might, by a less judicious observer, be mistaken for typhus; hence the necessity of the most accurate discrimination, since the mode of treatment, recommended perhaps too indiscriminately in typhus, is found highly injurious in this fever. With symptoms of the most extreme debility, are always associated either violent visceral inflammation, or great local congestion in some important organs. This might have been doubted, had not frequent dissections proved the fact beyond all question.

The period of the disease varies according to its violence. Death frequently occurs in the first week, and sometimes as early as the third day. In these cases the patient shows great anxiety, oppression of the breast, and laborious respiration. These symptoms rapidly increase with a pulse fluttering and sinking, or throbbing, till the last moment, which, under such circumstances, is seldom delayed longer than from six to twelve hours. The most favorable prognostic seems to have been formed when the pains, though violent, were shifting, and often in the extremities, and also when the disease assumed an intermittent form; but when relief follows the occurrence of dysenteric symptoms, the issue was generally fatal.

Before entering into a detail of the cure, Dr. Sutton premises the following remarks:

"In one instance of the occurrence of this disease, when treated as typhus, out of thirty-seven patients received into the hospital, eleven died.

"In another, where the same treatment was pursued upon a moderated plan (that is to say, without pressing the use of the bark, opium, wine, &c. in the early stage of the disease) out of ninety-two patients eighteen died.

"In another, in which the disease was treated as synochus, where moderate bleeding and evacuants were employed in the beginning of the disease, and the usual remedies for typhus were afterwards resorted to, the mortality was upon the average of three in twenty.

"By the treatment in which venesection has been relied on as a principal remedy, the greatest average of deaths, in any of the instances in which that plan of cure has been adopted, does not exceed one in twenty.

"In the above cited examples of the comparative fatality of this fever, the disease in each appeared to be in an aggravated form, and, so far as could be perceived, the cases were equal in violence, violence. Under each method of cure, therefore, at many times when this fever occurs, the mortality may be much less considerable. Out of seventy apparently severe cases of the disease received into the hospital at the same time, in which the remedies, that will be pointed out as procuring the most effectual relief, were adopted, every patient recovered. And it has been observed, that the disease has sometimes been attended with less fatality, when treated as typhus, than could possibly be expected, considering its inflammatory nature, which is clearly evinced by the appearances in the body after death.

"Nor ought it to be considered as a matter of little importance, in recommendation of the plan of cure by venesection, &c. that, in five cases in six, the patients become convalescent within the first week of the fever, with their strength but rarely impaired, the appetite generally greater than it is prudent to indulge, and their progress to perfect health seldom becomes protracted by any remaining effects of the previous disease."

The remedy principally relied on, was, the reader will perceive, blood-letting, which in one instance that proved favorable, was carried as far as eighty ounces. In other cases a single venesection, to the amount of thirty ounces of blood, proved a cure.

We have made these free extracts from this short work, just to impress our readers with a proper sense of its importance. The whole is written with the greatest care, and so much pains are taken to suppress every unnecessary remark, that it is impossible for us to convey the author's meaning in fewer words than he has used.

Excepting in the judicious writings of Dr. Robert Jackson, we have no where met with any account of so free an use of the lancet in fevers arising from contagion, and attended with such symptoms of debility. We cannot however for a moment question the propriety of the practice, and sincerely hope that the concurrence of two physicians, each of whom have had such large opportunities of ascertaining the fact, will be a means of rescuing many of the brave defenders of their country from an inglorious death, and preserve them for those services in which they are so much required.

---

*An Answer to Dr. Moseley, containing a Defence of Vaccination;* by John Ring, Member of the Royal College of Surgeons in London, &c. Octavo, pp. 290, with an Index. London, 1805.

This universal champion of vaccination, who declines no challenge, has treated his present antagonist with far greater attention than any other opponent of the new practice. As Mr. R. always appears to elaborate his defence in proportion to the respectability of his adversary, Dr. M. will find himself placed in this answer at an immense distance before all his competitors for the honour of impeding the Jennerian discovery. In the case of common opponents nents, or mere calumniators, Mr. R. contents himself with simple refutation, and dismisses them as concisely as possible; but Dr. M. is honoured with a pamphlet of 290 pages, in which his wit is answered by wit, his arguments by arguments, and even his uncandid statements are treated with considerable respect. In fact, Mr. R. appears to have availed himself of the rank Dr. M. holds in the profession, and the important public situation he fills, to give, thro' him, a general and full answer to all the *alleged* failures, consequent diseases, eruptions, &c. which have been so often, so industriously, and so unfairly (to say the least) dragged into public notice.

In our opinion Mr. R. has not failed in any part of his object; but, it has been observed in all ages that truth, philanthropy, and science cannot oppose falsehood, self interest and ignorance, on equal terms. The number of readers and their motives are most disproportionate. Those who read Dr. M's attack with pleasure, will not read Mr. R's answer with candour; this, however, is the lot of humanity, and arises from causes that will never cease to operate.

We have no doubt that all the friends of vaccination will cordially thank their indefatigable friend and champion; and if they read this Answer with the same pleasure that it has afforded us, they will confess that superior gratifications arising from controversy, so conducted, are rare indeed. To us, Mr. R's answer appears to be as complete a refutation of all the reasoning, as satisfactory a reply to all the alleged cases, and as brilliant a retort of all the wit, as such an attack can require.

Mr. R. has divided his answer into several parts or sections; the first, to page 19, contains general observations on the style, manner, and spirit of Dr. M's attack. In the next, he examines the first part of Dr. M's Treatise at considerable length, and proves that the inoculation of the small-pox, on its first introduction into England, met with infinitely more opposition than vaccination has encountered. That this opposition was far more general and inveterate than the present, and supported by characters of much importance in society.

Our author then adverts to the London testimonial in favour of vaccination, to the number and respectability of the signatures, and contrasts them with the opponents of the practice. This leads him to the evidence given before the Committee of the House of Commons. The enemies of Dr. Jenner's discovery never appear to recollect, that the greatest care was taken by him that all his acknowledged opponents should be personally examined by the Committee, and all unfavourable reports, however frivolous, properly investigated.

After this inquisition, examination, and investigation, the House of Commons came to the resolution which planted such rankling thorns in the breasts of Dr. Jenner's enemies. This decision, together with the rapid establishment of the Royal Jennerian Society,

Society, silenced as far as possible, all avowed opposition to the practice for a considerable time. It was not however in England alone that the importance of vaccination was duly appreciated, for an institution similar to the Royal Jennerian Society was established in Paris, and a regular correspondence with the other departments of the empire provided for. Mr. R, has given the plan of this institution, and subjoined an account of the progress of vaccination in Germany and other parts of the Continent.

After some criticisms on other publications, our author gives an account of various instances in which the small-pox occurred a second time, as well after the natural as the inoculated disease; instances of the latter kind are, indeed, far from uncommon.

As the cases in Fullwood's Rents produced a stronger sensation in the public mind, on account of the great number of practitioners who witnessed them, and the candid investigation of them by the Committee, than any other imputed failures; so Mr. R. has been more minute and circumstantial in his examination of the weight they ought to have with the public. In this, however, as well as other parts of his Answer, a number of collateral topics are frequently introduced, which, though connected with the subject, sometimes distract the attention, and always diminish the weight of the principal argument; but as the attack was desultory, so is the answer. The question at issue is of the first importance, and consequently it required a full and extensive discussion to establish the truth, and expose misrepresentation; but this very copiousness is in danger of injuring the cause it ought to serve, for many will read a short pamphlet, that abounds with assertion, invective, abuse, calumny or misrepresentation, on a popular subject, while very few will have perseverance enough to weigh and compare the reasoning of a voluminous answer. In this respect, as we have already said, truth, philanthropy and candour are very unequally opposed to falsehood, bigotry and misrepresentation; and on this account we think the size of Mr. Ring's book will considerably diminish the good it is otherwise so well calculated to produce.

